# Associations Among Diet Quality, Motor Competence, and Physical Fitness in Preschool Children: A Cross-Sectional Study

**DOI:** 10.3390/nu18152519

**Published:** 2026-08-03

**Authors:** Josivaldo de Souza-Lima, Andrés Godoy-Cumillaf, Frano Giakoni-Ramírez, Catalina Muñoz-Strale, Maribel Parra-Saldias, Daniel Duclos-Bastias, Claudio Farias-Valenzuela, Eugenio Merellano-Navarro, José Bruneau-Chávez

**Affiliations:** 1 Facultad de Educación y Humanidades, Escuela de Ciencias del Deporte, Universidad Andres Bello, Las Condes, Santiago 7550000, Chile; josivaldo.desouza@unab.cl (J.d.S.-L.); frano.giakoni@unab.cl (F.G.-R.); catalina.munoz@unab.cl (C.M.-S.); 2 Grupo de Investigación en Educación Física, Salud y Calidad de Vida (EFISAL), Facultad de Educación, Universidad Autónoma de Chile, Temuco 4780000, Chile; 3 Departamento de Educación Física, Deporte y Recreación, Universidad de Atacama, Copiapó 1530000, Chile; maribel.parra@uda.cl; 4 iGEO, Escuela de Educación Física, Facultad de Filosofía y Educación, Pontificia Universidad Católica de Valparaíso, Valparaíso 2340025, Chile; daniel.duclos@pucv.cl; 5 METIS Research Laboratory, Facultad de Negocios y Tecnología, Universidad Alfonso X el Sabio (UAX), 28691 Madrid, Spain; 6 Escuela de Ciencias de la Actividad Física, Universidad de Las Américas, Santiago 1530000, Chile; claudio.farias.valenzuela@edu.udla.cl; 7 Department of Physical Activity Sciences, Faculty of Education Sciences, Universidad Católica del Maule, Talca 3530000, Chile; emerellano@ucm.cl; 8 Departamento de Educación Física, Deportes y Recreación, Universidad de la Frontera, Temuco 4811230, Chile; jose.bruneau@ufrontera.cl

**Keywords:** postural stability, early childhood, cardiorespiratory fitness, movement skills, child development

## Abstract

Background/Objectives: Diet quality, motor competence, and physical fitness are important domains of child development, yet their interrelationships during the preschool years remain insufficiently understood. This study examined whether diet quality was independently associated with motor competence and physical fitness and explored the potential role of motor competence and postural stability within these relationships in preschool children. Methods: A cross-sectional study was conducted in 134 preschool children aged 4–6 years. Motor competence was assessed using selected tasks from the Movement Assessment Battery for Children-2, and physical fitness was evaluated using tests from the PREFIT battery and the sit-and-reach test. Diet quality was estimated using a food frequency questionnaire from which a Diet Quality Index (DQI) was derived. Multiple linear regression models adjusted for age, sex, and body mass index were used to examine the associations among study variables. Exploratory pathway analyses and prespecified sensitivity analyses were additionally performed. Results: Diet quality showed a positive but non-significant association with motor competence, whereas no significant direct association with physical fitness was observed after adjustment for age, sex, and body mass index. Exploratory pathway analyses likewise did not provide evidence of statistically significant indirect associations between diet quality and physical fitness through motor competence, although the direction of the observed associations was consistent across sensitivity analyses. In addition, higher Global Motor Competence and Postural Stability were consistently associated with better global physical fitness, lower-limb muscular strength, cardiorespiratory fitness, flexibility, and speed/agility. Conclusions: The present study found no evidence of significant direct or indirect associations between diet quality and physical fitness after multivariable adjustment, although healthier dietary patterns showed consistently favorable trends. Consistent with previous evidence, motor competence, particularly postural stability, was positively associated with multiple components of physical fitness.

## 1. Introduction

Early childhood is a critical developmental period characterized by rapid physical, motor, cognitive, and behavioral changes that may influence health trajectories throughout childhood and adolescence [1,2]. During the preschool years, children acquire and refine fundamental movement skills, develop movement-related capacities such as balance and coordination, and establish behavioral patterns related to physical activity, sleep, and diet that may shape later development [3,4,5]. Accordingly, identifying factors associated with healthy physical development during this stage has become a priority in pediatric health research [4,6].

Physical fitness is being increasingly recognized as an important marker of current and future health in children [7,8]. Components such as cardiorespiratory fitness, muscular strength, flexibility, and speed/agility have been associated with favorable adiposity profiles, cardiometabolic health, skeletal health, and selected aspects of psychological well-being in pediatric populations, although the strength of evidence varies across outcomes [7,9,10,11]. Therefore, understanding the factors associated with physical fitness during the preschool years is essential for informing more effective strategies for early health promotion [8,11].

Among the behavioral factors potentially associated with physical fitness, diet quality has received growing attention [12]. Healthy dietary patterns characterized by higher consumption of fruits, vegetables, legumes, fish, and other nutrient-dense foods have been linked to better cognition, academic achievement, and neurodevelopment in children [12,13,14]. In contrast, dietary patterns characterized by frequent consumption of processed or ultra-processed foods, saturated fats, and sugars have been associated with poorer cognitive and neurodevelopmental outcomes, and early-life exposure to these patterns appears unfavorable for brain development [13,15,16]. However, although diet quality is broadly recognized as an important determinant of child health, the extent to which it is associated with physical fitness during the preschool years remains less clear. Current evidence indicates that associations with physical fitness appear to be modest and are likely to operate alongside multiple developmental and behavioral factors rather than through strong direct relationships [12].

Beyond behavioral factors, motor competence has emerged as one of the factors most consistently associated with physical fitness during early childhood [17,18]. Fundamental motor skills include domains such as locomotor, object control, and stability or balance skills, and these capacities are considered building blocks for later physical activity participation and broader physical development [6,19,20]. Previous evidence suggests that children with higher motor competence tend to show better physical fitness, and longitudinal data indicate a bidirectional predictive relationship between motor skills and fitness in preschoolers [6,20]. Collectively, these findings suggest that motor competence represents a key component of early childhood physical development and a functional domain closely associated with physical fitness. Therefore, further investigation is warranted to better understand how motor competence relates to other health-related factors, including dietary behaviors and diet quality, during this developmental period.

Within the broader construct of motor competence, postural stability may be particularly relevant during the preschool years. Balance and coordination are core motor domains in early childhood, and structured coordination training across the preschool period has been shown to improve balance regulation and postural control [1,20]. Shorter preschool interventions have also improved dynamic balance, static balance, and related coordination outcomes, supporting the relevance of balance-related performance as an informative motor domain in this age group [1,17,19]. Because postural stability underpins the efficient control of body position during both static and dynamic motor tasks [1,17,19], it may represent an informative component of motor competence when investigating factors associated with multiple components of physical fitness in preschool children. Recent evidence has further suggested that postural stability may be independently associated with several components of physical fitness during the preschool years [21], supporting its consideration as a distinct motor domain within the present study.

Despite growing interest in these domains, important gaps remain. Although the association between motor competence and physical fitness has been increasingly investigated, fewer studies have simultaneously examined diet quality, motor competence, and physical fitness within the same sample of preschool children [6,12,20]. Furthermore, although healthier dietary patterns have been associated with multiple aspects of child development, relatively little is known about whether diet quality is also associated with motor competence and whether these relationships are reflected in physical fitness outcomes during early childhood [12,13,14,20]. Addressing these questions may contribute to a more integrated understanding of the behavioral and functional factors associated with physical development during the preschool years.

Taken together, these considerations provide the rationale for the present study. Therefore, the aim of the present study was to examine whether diet quality was independently associated with motor competence and physical fitness and to explore the potential role of motor competence and postural stability in these associations among preschool children. We hypothesized that higher diet quality would be associated with better motor competence and physical fitness and that motor competence and postural stability would be associated with physical fitness in preschool children.

## 2. Materials and Methods

### 2.1. Study Design and Participants

This cross-sectional study examined the associations among diet quality, motor competence, postural stability, and physical fitness in preschool children aged 4–6 years. Participants were recruited between August and November 2024 from six early childhood education centers in the city of Temuco, Chile, comprising eight preschool classrooms. A convenience sampling approach was used, and all eligible preschool children attending the participating centers during the recruitment period were invited to participate. Information was collected on anthropometric characteristics, motor competence, physical fitness, and dietary habits. All assessments were performed by trained evaluators following standardized testing procedures.

The study was conducted in accordance with the ethical principles for research involving human participants and adhered to the Declaration of Helsinki. Written informed consent was obtained from parents or legal guardians before participation, and verbal assent was requested from all children prior to the assessments. The study protocol was reviewed and approved by the Ethics Committee of Universidad Autónoma de Chile (CEC 31-22), with ethical approval granted on 8 August 2022.

A total of 750 preschool children were invited to participate in the study. Of these, 496 children (66.1%) provided parental consent and completed the study assessments. Children were eligible if they were between 4 and 6 years of age, enrolled in one of the participating early childhood education centers, and had written parental consent and given their own assent. Participants with incomplete dietary, motor competence, physical fitness, or anthropometric data required for the present analyses were excluded from the corresponding analytical models.

The primary regression analyses were based on complete-case data. The reduction in the analytical sample was primarily attributable to incomplete dietary questionnaires and missing information for one or more variables required in the multivariable regression analyses. Accordingly, the principal regression, pathway, and sensitivity analyses were performed using data from 134 participants with complete information for the corresponding variables.

The present study was conducted within a broader research project involving 496 preschool children from Temuco, Chile. This research program was designed to investigate multiple dimensions of preschool health, including dietary habits, motor competence, postural stability, physical fitness, and anthropometric characteristics. Different analytical investigations were subsequently developed to address distinct research questions using the variables required for each objective. Because all studies originated from the same broader research project, some participant overlap across analytical investigations may have occurred. However, the present study was specifically designed to examine the associations among diet quality, motor competence, postural stability, and physical fitness. The dietary variables analyzed in this study, including the Food Frequency Questionnaire, Healthy Food Score (HFS), Unhealthy Food Score (UFS), and Diet Quality Index (DQI), have not been previously reported. Accordingly, the present study represents an independent scientific investigation addressing a novel research question rather than a secondary analysis of previously reported findings. A detailed comparison between the present study and the authors’ previous publication is provided in Supplementary Table S1.

**Figure 1:**
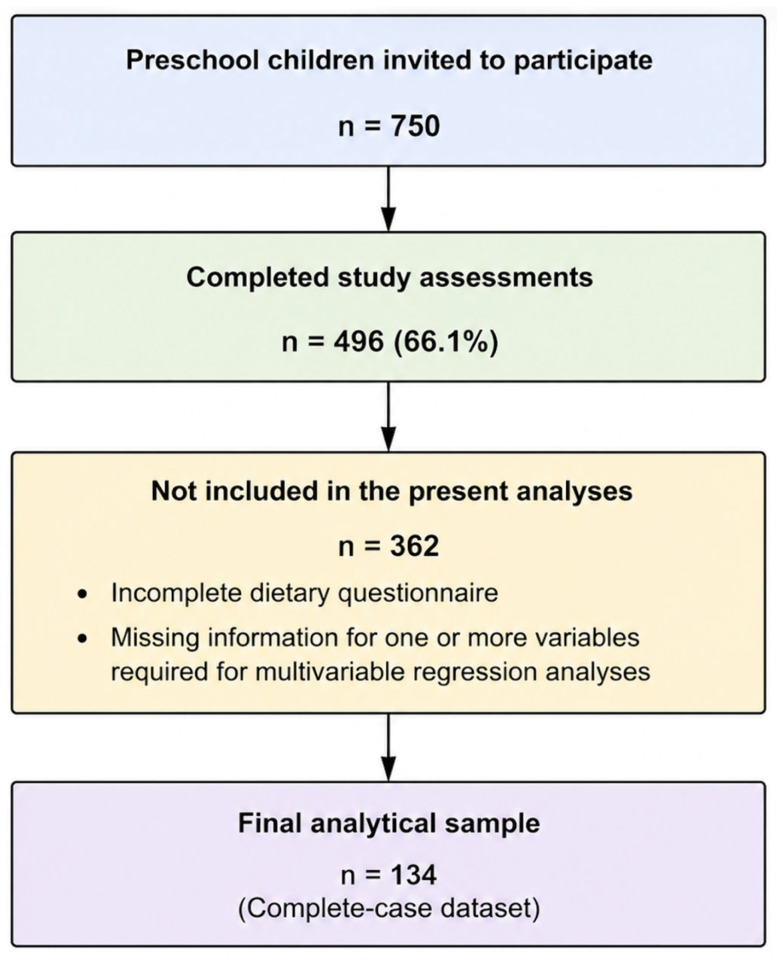
Participant flow diagram.

A flow diagram summarizing participant recruitment, enrolment, and selection of the analytical sample is presented in Figure 1.

### 2.2. Anthropometric Assessment

Body weight, height, and waist circumference were measured following standardized anthropometric procedures. Body mass index (BMI) was subsequently calculated as body weight in kilograms divided by height in meters squared (kg/m^2^). These anthropometric variables were used to characterize the study sample, while BMI was included as an adjustment variable in the statistical models.

Age, sex, and BMI were selected a priori as covariates because they are well-established determinants of children’s growth, motor development, and physical fitness and were considered potential confounding variables in the associations examined in the present study. Adjusting for these variables allowed the associations between diet quality, motor competence, and physical fitness to be examined while accounting for differences related to growth and developmental status. BMI was included consistently across all adjusted regression models to maintain a common adjustment strategy and to account for differences in body size and developmental status that could influence the associations under investigation.

### 2.3. Motor Competence

Motor competence was assessed using selected tasks from the Movement Assessment Battery for Children, Second Edition (MABC-2) [22], a widely used instrument with adequate psychometric properties for assessing motor performance in children [23]. The assessment included two object-control tasks (catching and throwing) and four postural stability tasks (right-leg balance, left-leg balance, tiptoe walking, and balance mat performance).

Because the individual motor tasks were measured using different scales and score ranges, each task was first standardized (z-score) prior to the construction of composite variables. This procedure ensured that all tasks contributed equally to the composite scores regardless of their original measurement scale or variance.

A standardized Global Motor Competence score (GMC_z) was calculated as the mean of the standardized scores from all six motor tasks. In addition, two domain-specific standardized indicators were derived. Standardized Postural Stability (PS_z) was calculated as the mean of the standardized scores for right-leg balance, left-leg balance, tiptoe walking, and balance mat performance, representing both static and dynamic aspects of postural control. Standardized Object Control (OC_z) was calculated as the mean of the standardized scores for catching and throwing performance.

These standardized composite indicators were subsequently used to examine both global and domain-specific associations with physical fitness outcomes.

All motor competence assessments were administered by trained evaluators following the standardized procedures described in the MABC-2 manual. Standardized verbal instructions and demonstrations were provided before each task, and children were allowed practice trials when specified in the testing protocol. Performance was scored according to the standardized MABC-2 scoring criteria.

### 2.4. Physical Fitness

Physical fitness was assessed using field-based tests primarily derived from the PREFIT battery [24], an instrument that has demonstrated feasibility, reliability, and suitability for use in preschool children [25]. In addition, the PREFIT battery has recently shown good reliability in Chilean preschool populations [26].

The assessed components included lower-limb muscular strength, evaluated using the standing long jump test; cardiorespiratory fitness, assessed using the 20 m shuttle run test; and speed/agility, assessed using the 4 × 10 m shuttle run test. Flexibility was additionally assessed using the sit-and-reach test following standardized procedures. Cardiorespiratory fitness was expressed as the final completed stage of the 20 m shuttle run test, with higher stages indicating better cardiorespiratory fitness.

To obtain a standardized indicator of overall physical fitness, a Global Physical Fitness (GPF) score was calculated as the mean of the standardized scores for lower-limb muscular strength, flexibility, cardiorespiratory fitness, and speed/agility. Because lower times in the speed/agility test indicate better performance, speed values were inverted before standardization so that higher scores consistently reflected better physical fitness across all components.

All physical fitness assessments were conducted by trained evaluators according to the standardized PREFIT protocols. Participants received standardized verbal instructions before each test and were encouraged to perform maximally. The number of trials, scoring procedures, and rest intervals followed the recommendations of the PREFIT battery, with the best performance recorded for analysis when multiple trials were specified.

### 2.5. Diet Quality

Dietary habits were assessed using a Spanish-language food frequency questionnaire (FFQ) completed by parents or legal guardians. The questionnaire comprised 15 food groups commonly consumed during childhood (fruits, vegetables, sweets, sugar-sweetened beverages, diet/light beverages, semi-skimmed milk, whole milk, cheese, dairy products, cereals, white bread, whole-grain bread, chips, French fries, and fish) and evaluated the child’s habitual weekly consumption frequency for each food group. Parents selected one of seven response categories: never, less than once per week, once per week, 2–4 times per week, 5–6 times per week, once per day, or more than once per day. The complete questionnaire is provided in Supplementary Appendix SA1.

Food frequency questionnaires are widely used in pediatric nutritional epidemiology to characterize habitual dietary intake and dietary patterns, particularly when the objective is to evaluate overall diet quality rather than estimate absolute nutrient intake [27,28]. Similar approaches have been used in Spanish pediatric populations to investigate associations between dietary habits, adiposity, physical fitness, and other health-related outcomes [29,30,31].

Food groups were classified a priori according to their intended representation of healthier and less healthy dietary habits within the context of the questionnaire. The Healthy Food Score (HFS) included food groups considered to represent healthier dietary habits (fruits, vegetables, fish, whole-grain bread, semi-skimmed milk, and dairy products), whereas the Unhealthy Food Score (UFS) included food groups considered to represent less healthy dietary habits (sweets, sugar-sweetened beverages, chips, and French fries). Because the questionnaire assessed broad food groups rather than specific food products or processing characteristics, this classification was intended to reflect the overall nutritional profile of each food group within the context of the questionnaire.

Three composite dietary indicators were derived a priori from the FFQ. Each response category was coded on an ordinal scale from 1 to 7, reflecting increasing consumption frequency, and no reverse coding or weighting was applied. The Healthy Food Score (HFS) was calculated as the unweighted sum of six food groups considered to represent healthier dietary habits: fruits, vegetables, fish, whole-grain bread, semi-skimmed milk, and dairy products (theoretical range: 6–42). The Unhealthy Food Score (UFS) was calculated as the unweighted sum of four food groups representing less healthy dietary habits: sweets, sugar-sweetened beverages, chips, and French fries (theoretical range: 4–28). The Diet Quality Index (DQI) was subsequently calculated as the difference between the HFS and the UFS (DQI = HFS − UFS), yielding a theoretical range from −22 to 38, with higher values indicating better overall diet quality. The DQI was developed specifically for the present study as a composite dietary-frequency score intended to summarize the balance between healthy and unhealthy food consumption frequencies within the study population. Accordingly, it should be interpreted as an exploratory measure of relative dietary patterns rather than as a validated dietary quality index, as it does not account for portion size, total energy intake, or adherence to food-specific dietary recommendations. Although the FFQ included 15 food groups, only those considered a priori to represent healthy or unhealthy dietary patterns were included in the construction of the HFS and UFS, whereas the remaining food groups contributed to the overall dietary assessment but were not incorporated into the composite dietary indices. The food groups included in each dietary score and the corresponding scoring procedure are summarized in Supplementary Table S2.

The DQI was considered the primary dietary exposure because it provides an overall summary of the balance between healthy and unhealthy food consumption frequencies within the study population. As sensitivity analyses, the HFS and UFS were additionally examined separately to evaluate the robustness of the observed associations. No missing responses were present for the FFQ items in the analytical sample; therefore, all dietary scores were calculated using complete data.

### 2.6. Construction of Composite Variables

To represent broader functional domains and reduce data dimensionality, several composite variables were constructed prior to the statistical analyses. As described above, Global Motor Competence (GMC_z) was calculated as the mean of the standardized (z) scores from the six motor tasks, while Postural Stability (PS_z) and Object Control (OC_z) were calculated as the means of the standardized scores corresponding to their respective motor domains.

Global Physical Fitness (GPF) was calculated as the mean of the standardized scores for lower-limb muscular strength, cardiorespiratory fitness, speed/agility, and flexibility. Because lower times in the speed/agility test indicate better performance, speed/agility values were inverted prior to standardization so that higher scores consistently reflected better overall physical fitness.

These composite variables were used in the primary regression analyses, exploratory pathway analyses, and sensitivity analyses.

### 2.7. Statistical Analysis

Descriptive statistics were calculated to characterize the study sample. The distribution of continuous variables was assessed using the Shapiro–Wilk test together with visual inspection of histograms and quantile–quantile (Q–Q) plots. Continuous variables were summarized as means and standard deviations, whereas categorical variables were summarized as frequencies and percentages.

The primary analytical approach was based on multiple linear regression. Separate regression models were fitted to examine the associations of standardized Global Motor Competence (GMC_z) and standardized Postural Stability (PS_z) with physical fitness outcomes. The primary outcomes were Global Physical Fitness (GPF), Lower-Limb Muscular Strength, Cardiorespiratory Fitness, Flexibility, and Speed/Agility. Because GMC_z and PS_z represent related constructs, they were examined in separate adjusted models rather than entered simultaneously into the same model. All regression models were adjusted a priori for age, sex, and body mass index (BMI). Regression results are presented as unstandardized regression coefficients (B), standard errors (SEs), 95% confidence intervals (95% CIs), and corresponding *p*-values.

To further explore the statistical relationships among diet quality, motor competence, and physical fitness, exploratory pathway analyses were performed. These analyses evaluated the association between Diet Quality Index (DQI) and GMC_z, followed by the association between GMC_z and GPF after adjustment for DQI. In addition, an exploratory indirect effect was estimated using a non-parametric bootstrap procedure with 5000 resamples [32]. Because of the cross-sectional design, these analyses were interpreted as exploratory and hypothesis-generating rather than as evidence of causal mediation.

Sensitivity analyses were additionally performed using the Healthy Food Score (HFS) and Unhealthy Food Score (UFS) separately to evaluate the robustness of the associations observed with the Diet Quality Index [33]. These analyses followed the same adjustment strategy as the primary regression models.

Model assumptions were evaluated by examining residual normality, homoscedasticity, multicollinearity, and influential observations. Residual normality was assessed using the Shapiro–Wilk test together with visual inspection of Q–Q plots. Homoscedasticity was evaluated using the Breusch–Pagan test and inspection of residual-versus-fitted plots. Multicollinearity was assessed using variance inflation factors (VIF) [34], and influential observations were evaluated using Cook’s distance [35]. As an additional robustness analysis, heteroscedasticity-consistent HC3 standard errors were estimated [36]. No imputation procedures were applied, and all analyses were performed using complete-case data. Consequently, the effective sample size for the primary regression, pathway, and sensitivity analyses was 134 participants. Statistical significance was established at *p* < 0.05. Given the observational and cross-sectional nature of the study, all findings were interpreted as associative rather than causal.

All statistical analyses were performed using Python 3. Data management was conducted using pandas, regression analyses were performed using statsmodels, bootstrap analyses were implemented using statsmodels and custom resampling procedures, and graphical outputs were generated using matplotlib. Statistical computing was additionally supported by NumPy and SciPy.

## 3. Results

The descriptive characteristics of the complete-case study sample (*n* = 134) are presented in Table 1. The sample included preschool children aged 4–6 years, with a mean age of 5.33 ± 0.79 years and a mean body mass index of 17.63 ± 2.70 kg/m^2^. Girls represented 56.7% of the sample. Table 1 also summarizes the anthropometric characteristics, motor competence, physical fitness, and diet quality indicators of the study participants.

**Figure 2:**
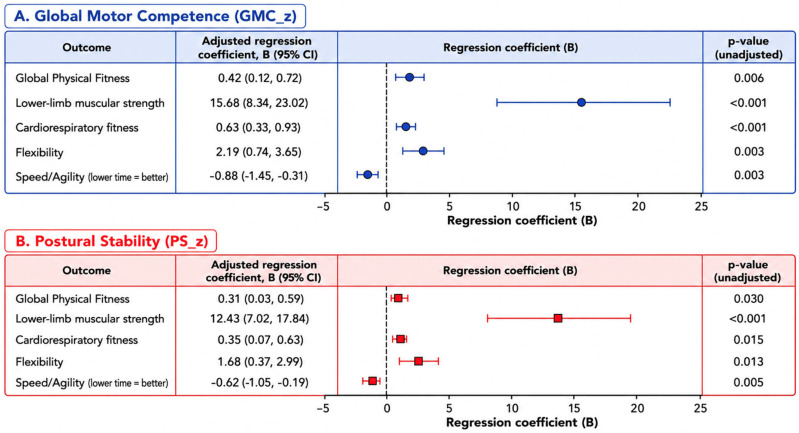
Adjusted associations of global motor competence (**A**) and postural stability (**B**) with physical fitness outcomes in preschool children. Adjusted regression coefficients (B), 95% confidence intervals (CI), and corresponding unadjusted *p*-values are presented for each adjusted linear regression mode. All regression models were based on 134 participants with complete data. Because outcomes are expressed in different measurement units, regression coefficients should be interpreted within each outcome and should not be compared directly. Across outcomes. Lower values in the Speed/Agility test indicate better performance. *p*-values are unadjusted for multiple comparisons. CI: confidence interval. GMC_z: Global Motor Competence (z-score). PS_z: Postural Stability (z-score).

Consistent with previous evidence, the adjusted associations between the standardized motor competence indices and physical fitness outcomes are presented in Table 2 and illustrated in Figure 2. After adjustment for age, sex, and body mass index (BMI), both Global Motor Competence (GMC_z) and Postural Stability (PS_z) were significantly associated with all physical fitness outcomes (all *p* < 0.05). Specifically, higher values of both indices were associated with better Global Physical Fitness, greater Lower-Limb Muscular Strength, better Cardiorespiratory Fitness, greater Flexibility, and better Speed/Agility performance. Detailed regression coefficients and corresponding confidence intervals are presented in Table 2.

The exploratory pathway analysis evaluating the association between diet quality and Global Physical Fitness through Global Motor Competence is presented in Supplementary Table S1, and the analytical framework is illustrated in Supplementary Figure S1. The Diet Quality Index (DQI) showed a positive, although non-significant, association with GMC_z (B = 0.016, *p* = 0.075). After adjustment for DQI, GMC_z remained positively associated with Global Physical Fitness (B = 0.537, *p* < 0.001). Neither the total nor the direct association between DQI and Global Physical Fitness reached statistical significance. Likewise, the estimated indirect effect of DQI through GMC_z was small (B = 0.009), and the bootstrap 95% confidence interval included zero (−0.001 to 0.020), indicating no statistically significant indirect association.

Sensitivity analyses using the Healthy Food Score (HFS) and Unhealthy Food Score (UFS) as alternative dietary exposures are presented in Supplementary Table S4. HFS showed positive, although non-significant, associations with both GMC_z and Global Physical Fitness. In contrast, UFS showed a non-significant inverse association with GMC_z, whereas no significant association was observed with Global Physical Fitness. Overall, these sensitivity analyses supported the robustness of the primary findings by showing consistent patterns across the alternative dietary indicators.

Regression diagnostic analyses generally supported the adequacy of the adjusted linear models (Supplementary Tables S5 and S6). Multicollinearity was negligible across all models, with maximum variance inflation factors ranging from 1.10 to 1.36. Residual-versus-fitted plots supported linearity, and Durbin–Watson statistics ranged from 1.67 to 2.05. Breusch–Pagan tests indicated heteroscedasticity in the cardiorespiratory fitness and flexibility models; however, sensitivity analyses using HC3 robust standard errors did not materially alter the magnitude, direction, or statistical significance of the associations. Influence analyses produced similar conclusions for most models. The association between process-oriented motor competence and speed/agility was the only exception, becoming non-significant after exclusion of seven potentially influential observations; this result should therefore be interpreted cautiously.

## 4. Discussion

The present study examined the associations among diet quality, motor competence, and physical fitness in preschool children, with particular emphasis on whether motor competence could represent a functional domain linking dietary behaviors with physical fitness. Three main findings emerged. First, although the Diet Quality Index (DQI) showed a positive tendency toward better motor competence, neither the direct nor the exploratory indirect associations between diet quality and physical fitness reached statistical significance. Second, sensitivity analyses using the Healthy Food Score (HFS) and Unhealthy Food Score (UFS) yielded directionally consistent findings, supporting the robustness of the primary analyses. Third, consistent with previous evidence, both Global Motor Competence (GMC_z) and Postural Stability (PS_z) were significantly associated with all measured components of physical fitness after adjustment for age, sex, and body mass index.

The absence of statistically significant associations between diet quality and physical fitness should not be interpreted as evidence that dietary behaviors are unrelated to early childhood physical development. Rather, the positive direction of the observed associations between the DQI and motor competence, together with the consistent findings obtained using alternative dietary indicators, suggests that diet quality may contribute to motor development, although the magnitude of these relationships was modest in the present sample. These findings should be interpreted cautiously given the cross-sectional design and the exploratory nature of the pathway analysis.

Within this broader developmental framework, motor competence emerged as a key correlate of physical fitness. Consistent with previous evidence, both GMC_z and PS_z were independently associated with all measured physical fitness components, reinforcing current developmental models that consider motor competence a fundamental determinant of children’s functional capacity during the preschool years [37,38,39,40]. Together, these findings support an integrated perspective in which healthy dietary behaviors and motor competence should be viewed as complementary, rather than isolated, contributors to children’s physical development.

Consistent with previous evidence, the present study confirmed a strong and consistent association between motor competence and physical fitness during the preschool years. After adjustment for age, sex, and body mass index, higher Global Motor Competence (GMC_z) was associated with better performance across all measured physical fitness components, including global physical fitness, lower-limb muscular strength, cardiorespiratory fitness, flexibility, and speed/agility. These findings are in agreement with current developmental models proposing reciprocal interactions between motor competence and physical fitness throughout childhood, whereby children with greater motor competence are more likely to engage successfully in movement experiences that further reinforce physical fitness and motor development [37,38,39,40,41]. Although the cross-sectional design precludes conclusions regarding temporal direction, the consistency of these associations further supports the close interrelationship between these domains during early childhood.

Postural stability also emerged as an important correlate of physical fitness, showing consistent associations with every physical fitness component evaluated. This finding is biologically plausible because efficient postural control provides the foundation for maintaining body alignment, coordinating movement, and generating force effectively during both static and dynamic motor tasks [38,42,43]. During the preschool years, when rapid neuromuscular maturation and sensory integration occur, adequate postural stability may facilitate the acquisition of increasingly complex motor skills and improve performance across multiple domains of physical fitness. These findings are also consistent with our previous study in preschool children, which demonstrated positive associations between postural stability and lower-limb muscular strength, speed/agility, and cardiorespiratory fitness [21]. The present study extends those observations by showing that postural stability was consistently associated with all measured physical fitness components, including global physical fitness and flexibility, further supporting its value as an indicator of children’s functional capacity during early childhood.

Although diet quality was not significantly associated with physical fitness in the present study, the observed pattern of results suggests that dietary behaviors may still contribute to early childhood physical development through mechanisms that warrant further investigation. Children with healthier dietary patterns tended to exhibit better motor competence, although this association did not reach statistical significance. Likewise, neither the direct association between the Diet Quality Index (DQI) and Global Physical Fitness nor the exploratory indirect association through motor competence was statistically significant. Therefore, the present findings do not support a mediating role of motor competence in the relationship between diet quality and physical fitness. Instead, they should be interpreted as preliminary evidence that may help guide future longitudinal and intervention studies designed to clarify these complex developmental relationships. Because all variables were measured at a single time point, the proposed sequence linking diet quality, motor competence, and physical fitness should be interpreted as a conceptual framework for hypothesis generation rather than as evidence of temporal or causal relationships.

Despite the absence of statistically significant associations, the direction of the observed relationships is consistent with previous evidence indicating that healthier dietary patterns support multiple aspects of child development, including growth, neurodevelopment, body composition, and physical functioning [27,44]. Adequate nutrition during the preschool years provides essential substrates for musculoskeletal development, neuromuscular maturation, and central nervous system development, all of which contribute to the acquisition and refinement of motor skills. However, these developmental processes are influenced by numerous biological, behavioral, environmental, and social factors, which may partly explain why the associations observed in the present cross-sectional study were relatively modest. Consequently, future prospective cohort studies and well-designed intervention trials incorporating repeated assessments of dietary quality, motor competence, and physical fitness will be essential to determine whether improvements in diet quality contribute to motor development and, ultimately, to better physical fitness throughout early childhood [27,44].

The present findings have several practical implications for research and early childhood health promotion. Although diet quality was not significantly associated with physical fitness in the present study, promoting healthy dietary habits during the preschool years remains a fundamental public health priority because of their well-established benefits for growth, neurodevelopment, and long-term health [27,44]. At the same time, the consistent associations observed between motor competence and physical fitness suggest that motor competence assessments may provide valuable information about children’s functional development and complement physical fitness evaluations during the preschool years. In particular, the inclusion of postural stability assessments within preschool health and educational settings may help identify children who could benefit from targeted interventions aimed at improving movement competence and overall physical functioning. Together, these findings support integrated strategies that simultaneously promote healthy nutrition and motor development during early childhood.

The present study has several strengths that should be considered when interpreting the findings. It simultaneously examined diet quality, motor competence, postural stability, and physical fitness within the same sample of preschool children using standardized assessments, composite indices of motor competence, and adjusted analytical models. Regression diagnostic and sensitivity analyses further supported the robustness of the adjusted regression models, with heteroscedasticity-consistent (HC3) standard errors confirming the primary findings across all models except for the association between process-oriented motor competence and speed/agility, which should be interpreted with caution due to its sensitivity to influential observations (Supplementary Tables S5 and S6). In addition to evaluating associations among these domains, the study incorporated exploratory pathway analyses and sensitivity analyses using alternative dietary indices, providing complementary information regarding the robustness and consistency of the primary findings. Furthermore, by focusing on preschool children, the study contributes to the literature on a developmental period in which evidence regarding the interplay among nutrition, motor competence, and physical fitness remains relatively limited [38,39,40].

Nevertheless, several limitations should be acknowledged. First, the cross-sectional design precludes conclusions regarding temporal relationships, causality, or mediation. In addition, reverse causality cannot be excluded, as motor competence, dietary behaviors, and physical fitness may influence one another over time. Second, although the sample size was sufficient for the planned analyses, the study was conducted in preschool children from a single geographic region of Chile, which may limit the generalizability of the findings to other populations. In addition, although participants were recruited from six early childhood education centers comprising eight preschool classrooms, clustering by center or classroom was not explicitly modelled. Therefore, some degree of within-center or within-classroom correlation cannot be completely excluded. Third, dietary quality was assessed using a parent-reported questionnaire and may therefore be subject to reporting bias. In addition, the Diet Quality Index used in the present study was a study-specific composite dietary-frequency score derived from the balance between healthy and unhealthy food consumption frequencies. Consequently, it did not account for portion sizes, total energy intake, or adherence to food-specific dietary recommendations and should therefore be interpreted as a relative indicator of dietary patterns rather than a comprehensive measure of overall diet quality. In addition, several potentially relevant confounding variables, including objectively measured physical activity, socioeconomic characteristics, parental education, sleep habits, screen time, outdoor play, and total energy intake, were not available and therefore could not be considered in the analyses. Consequently, residual confounding cannot be excluded, and the observed associations involving diet quality should be interpreted with appropriate caution. Finally, although the exploratory pathway analysis provided useful information for generating hypotheses, it should not be interpreted as evidence of mediation or causal mechanisms. Future longitudinal and intervention studies incorporating objective measures of health behaviors and repeated assessments of motor competence and physical fitness will be essential to clarify the developmental relationships observed in the present study.

## 5. Conclusions

The present study found no evidence of significant direct or exploratory indirect associations between diet quality and physical fitness in preschool children. Nevertheless, healthier dietary patterns showed directionally favorable associations with motor competence across both the primary and sensitivity analyses, suggesting that diet quality may contribute to early childhood motor development through mechanism and relationship that warrant further investigation. In agreement with previous evidence, both motor competence and especially postural stability were consistently associated with multiple components of physical fitness, reinforcing their importance as key domains of children’s functional development. Overall, these findings support an integrated approach to early childhood health that considers nutrition, motor competence, and physical fitness as interconnected domains while highlighting the need for longitudinal and intervention studies to clarify their temporal relationships and underlying mechanisms.

## Figures and Tables

**Table 1:** Descriptive characteristics of the study sample.

Variable	Total (Mean ± SD)
General characteristic and anthropometry	
Age (years)	5.33 ± 0.79
Sex	Girls: 76 (56.7%); Boys: 58 (43.3%)
Height (cm)	115.62 ± 9.61
Weight (kg)	23.62 ± 5.31
Body mass index (kg/m^2^)	17.63 ± 2.70
Waist circumference (cm)	56.64 ± 6.36
Motor competence	
Catching (n)	7.79 ± 2.45
Throwing (n)	4.11 ± 1.99
Right-leg balance (s)	16.53 ± 10.11
Left-leg balance (s)	17.14 ± 10.68
Tiptoe walking (n)	13.48 ± 3.71
Balance mat (n)	4.68 ± 0.69
Global Motor Competence z-score (GMC_z)	0.00 ± 0.64
Postural stability z-score (PS_z)	0.00 ± 0.72
Object control z-score (OC_z)	0.00 ± 0.77
Physical fitness	
Lower-limb muscular strength (cm)	79.70 ± 21.97
Flexibility (cm)	30.55 ± 5.27
Speed/agility (s)	17.20 ± 1.86
Cardiorespiratory fitness (stages)	1.72 ± 0.89
Global physical fitness (GPF)	0.00 ± 0.75
Diet quality	
Healthy Food Score (HFS)	23.67 ± 4.59
Unhealthy Food Score (UFS)	11.22 ± 3.32
Diet Quality Index (DQI)	12.45 ± 5.54

Data are presented as mean ± standard deviation unless otherwise indicated. Sex is presented as n (%). Raw Speed/Agility values are presented in seconds, with lower values indicating better performance.

**Table 2:** Adjusted associations of standardized global motor competence and postural stability with physical fitness outcomes in preschool children.

Outcome	Predictor	B	SE	95% CI Lower	95% CI Upper	*p*-Value
Global physical fitness	GMC_z	0.534	0.083	0.370	0.699	<0.001
	PS_z	0.419	0.076	0.268	0.570	<0.001
Lower-limb muscular strength	GMC_z	16.054	2.587	10.936	21.172	<0.001
	PS_z	12.769	2.359	8.101	17.436	<0.001
Cardiorespiratory fitness	GMC_z	0.455	0.110	0.239	0.672	<0.001
	PS_z	0.363	0.098	0.168	0.557	<0.001
Flexibility	GMC_z	1.701	0.771	0.176	3.225	0.029
	PS_z	1.372	0.685	0.016	2.728	0.047
Speed/agility	GMC_z	−1.062	0.239	−1.534	−0.590	<0.001
	PS_z	−0.790	0.216	−1.217	−0.362	<0.001

Values are unstandardized regression coefficients (B) with standard errors (SEs), 95% confidence intervals (CIs), and *p*-values obtained from separate multiple linear regression models. All models were adjusted for age, sex, and body mass index (BMI). Higher Global Physical Fitness (GPF) values indicate better overall physical fitness. Speed/Agility values are presented as raw seconds; lower values indicate better performance.

## Data Availability

The data presented in this study are available on request from the corresponding author. The data are not publicly available due to ethical standards.

## Supplementary Materials

The following supporting information can be downloaded at https://www.mdpi.com/article/10.3390/nu18152519/s1, Table S1: Comparison of the objectives, analytical approach, and scientific contribution of the present study and the authors’ previous publication; Appendix SA1: Spanish-language Food Frequency Questionnaire used for dietary assessment; Table S2: Food groups included in the Healthy Food Score (HFS), Unhealthy Food Score (UFS), and Diet Quality Index (DQI), together with the scoring procedure; Table S3: Exploratory pathway analysis evaluating the association between diet quality, motor competence and global physical fitness; Table S4: Sensitivity analysis evaluating the association of the diet quality index, healthy food score, and unhealthy food score with global motor competence and global physical fitness; Table S5: Model performance and diagnostic statistics for the adjusted multiple linear regression models; Table S6: Sensitivity analyses of adjusted multiple linear regression models using HC3 robust standard errors and influential-observation diagnostics; Figure S1: Analytical framework of the exploratory pathway analysis examining the statistical relationships among diet quality, motor competence, and global physical fitness; STROBE Statement—Checklist of items that should be included in reports of cross-sectional studies.

## Abbreviations

The following abbreviations are used in this manuscript:

BMI	Body Mass Index
CI	Confidence Interval
DQI	Diet Quality Index
FFQ	Food Frequency Questionnaire
GMC_z	Global Motor Competence
GPF	Global Physical Fitness
HFS	Healthy Food Score
MABC-2	Movement Assessment Battery for Children, Second Edition
OC_z	Object Control
PS_z	Postural Stability
SD	Standard Deviation
UFS	Unhealthy Food Score
SE	Standard errors
