# Supplementary material for: Associations Among Diet Quality, Motor Competence, and Physical Fitness in Preschool Children: A Cross-Sectional Study
Source: Nutrients. 2026 Aug 3;18(15):2519. doi: 10.3390/nu18152519 (PMC13468173; doi:10.3390/nu18152519)
Supplement: Supplementary file 1 [file nutrients-18-02519-s001.zip › nutrients-4418306-supplementary.pdf]

Table S1. Comparison of the objectives, analytical approach, and scientific contribution of the present study and the authors' previous publication.

Characteristic	Previous publication https://doi.org/10.3390/children13070910	Present study
Primary objective	To examine the association between postural stability and components of physical fitness in preschool children.	To examine the associations among diet quality, motor competence, and physical fitness in preschool children.
Primary exposure	Postural Stability Index (PSI).	Diet Quality Index (DQI), with complementary analyses using the Healthy Food Score (HFS) and Unhealthy Food Score (UFS).
Main outcomes	Lower-limb muscular strength, speed/agility, cardiorespiratory fitness, and flexibility.	Global motor competence, postural stability, object control, and global physical fitness.
Main research question	Whether postural stability is independently associated with different components of physical fitness.	Whether diet quality is associated with motor competence and physical fitness in preschool children.
Role of motor competence	Postural stability was the principal independent variable, whereas object-control skills were included as an adjustment variable.	Motor competence was one of the principal outcomes, evaluated through global and domain-specific composite indicators.
Dietary assessment	Not assessed.	Assessed using a Food Frequency Questionnaire (FFQ), from which the Healthy Food Score (HFS), Unhealthy Food Score (UFS), and Diet Quality Index (DQI) were derived.
Physical fitness	Primary outcome.	Included as a complementary outcome within the broader analytical framework examining diet quality and motor competence.
Main statistical analyses	Multiple linear regression, ANCOVA, and sensitivity analyses.	Multiple linear regression, exploratory pathway analysis, sensitivity analyses, and model diagnostics.
Principal contribution	Demonstrated associations between postural stability and selected components of physical fitness.	Extends previous work by incorporating diet quality as the primary exposure and simultaneously examining its associations with motor competence and physical fitness.
Scientific novelty	Focused on the relationship between motor performance and physical fitness.	Integrates nutritional, motor, and physical fitness domains within a single analytical framework.

DQI, Diet Quality Index; FFQ, Food Frequency Questionnaire; HFS, Healthy Food Score; UFS, Unhealthy Food Score; PSI, Postural Stability Index.

FRECUENCIA DE ALIMENTOS DEL NIÑO/A

¿Cuántas veces por semana suele su hijo/a comer o beber los siguientes alimentos?:

POR FAVOR, MARQUE CON UNA "X" <u>SÓLO UNA RESPUESTA EN CADA FILA</u>.

	Nunca	Menos de 1 vez por semana	1 vez por semana	2-4 veces por semana	5-6 veces por semana	1 vez al día, cada día	Más de 1 vez al día, cada día
**Fruta**							
**Verdura**							
**Dulces** (caramelos o chocolate)							
**Refrescos de cola u otros tipos que contengan azúcar**							
**Refrescos de cola light u otros tipos que sean light**							
**Leche desnatada o semidesnatada**							
**Leche entera**							
**Queso**							
**Otros productos lácteos** (yogur, batidos, flanes, natillas, etc.)							
**Cereales** (copos de maíz, muesli, cereales con chocolate, etc.)							
**Pan blanco**							
**Pan integral**							
**Chips**							
**Patatas fritas**							
**Pescado**							

Supplementary appendix SA1. Spanish-language Food Frequency Questionnaire used for dietary assessment.

Table S2. Food groups included in the Healthy Food Score (HFS), Unhealthy Food Score (UFS), and Diet Quality Index (DQI), together with the scoring procedure.

Food group	Included in HFS	Included in UFS
Fruits	✓	
Vegetables	✓	
Fish	✓	
Whole-grain bread	✓	
Semi-skimmed milk	✓	
Dairy products	✓	
Sweets		✓
Sugar-sweetened beverages		✓
Chips		✓
French fries		✓

Response categories were coded from 1, indicating the lowest consumption frequency, to 7, indicating the highest consumption frequency. The HFS and UFS were calculated as the unweighted sums of the corresponding food-group scores. The DQI was calculated as HFS − UFS, with higher values indicating better overall diet quality.

Table S3. Exploratory pathway analysis evaluating the association between diet quality, motor competence and global physical fitness.

Pathway	B	SE	95% CI	p-value
DQI → GMC_z	0.016	0.009	-0.002 to 0.034	0.075
GMC_z → GPF (adjusted for DQI)	0.537	0.085	0.370 to 0.705	<0.001
Total effect: DQI → GPF	0.007	0.010	-0.013 to 0.027	0.496
Direct effect (DQI → GPF adjusted for GMC_z)	-0.002	0.009	-0.020 to 0.016	0.826
Indirect effect: DQI → GMC_z → GPF	0.009	0.005	-0.001 to 0.020	0.065

Values are unstandardized regression coefficients (B) with standard errors (SE), 95% confidence intervals (CI), and p-values. The indirect effect was estimated using a non-parametric bootstrap procedure with 5,000 resamples. All regression models were adjusted for age, sex, and body mass index (BMI). Given the cross-sectional design, results should be interpreted as exploratory and hypothesis-generating rather than evidence of causal mediation.

Table S4. Sensitivity analysis evaluating the association of the diet quality index, healthy food score, and unhealthy food score with global motor competence and global physical fitness.

Exposure	Outcome	B	SE	95% CI	p-value
Diet Quality Index (DQI)	GMC_z	0.016	0.009	-0.002 to 0.034	0.075
Healthy Food Score (HFS)	GMC_z	0.013	0.011	-0.009 to 0.034	0.252
Unhealthy Food Score (UFS)	GMC_z	-0.021	0.015	-0.051 to 0.010	0.178
Diet Quality Index (DQI)	GPF	0.007	0.010	-0.013 to 0.027	0.496
Healthy Food Score (HFS)	GPF	0.012	0.012	-0.012 to 0.036	0.316
Unhealthy Food Score (UFS)	GPF	0.005	0.017	-0.029 to 0.038	0.788

Values are unstandardized regression coefficients (B) with standard errors (SE), 95% confidence intervals (CI), and p-values obtained from separate multiple linear regression models. All models were adjusted for age, sex, and body mass index (BMI).

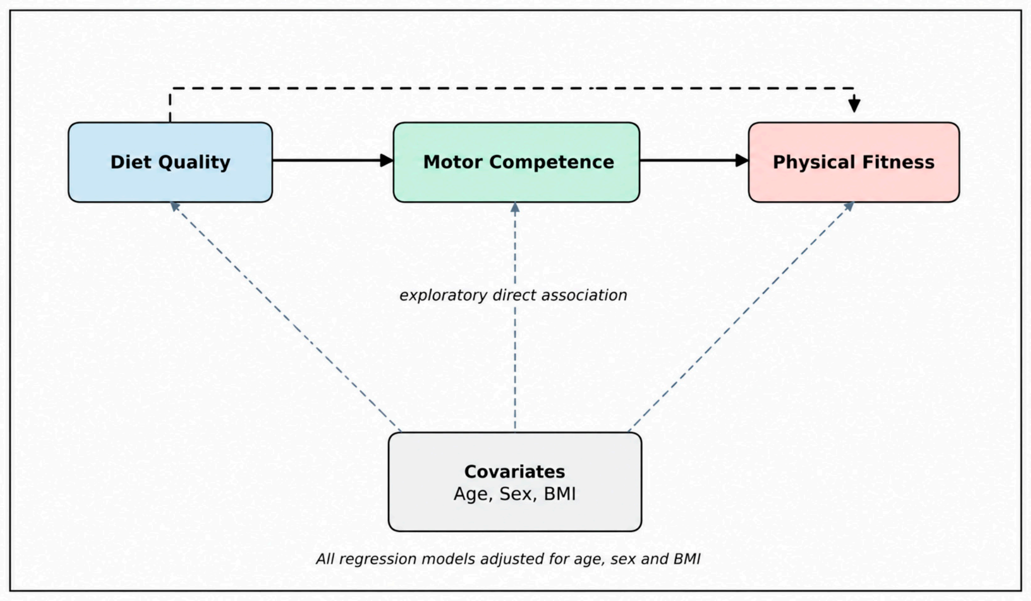

Figure S1. Analytical framework of the exploratory pathway analysis examining the statistical relationships among diet quality, motor competence, and global physical fitness. Analytical framework of the revised analytical model. Solid arrows indicate the primary hypothesized associations between Diet Quality, Motor Competence, and Physical Fitness. The dashed arrow represents the exploratory direct association between Diet Quality and Physical Fitness. All regression models were adjusted for age, sex, and body mass index (BMI).

Table S5. Model performance and diagnostic statistics for the adjusted multiple linear regression models.

Model	R^2	Adjusted R^2	Maximum VIF	Breusch–Pagan **p**	Residual assessment	Maximum Cook's distance	Cook's D > 4/**n** (n)
GPF ~ GMC_z	0.499	0.484	1.35	0.335	No important departure	0.110	7
LLMS ~ GMC_z	0.430	0.412	1.35	0.367	Mild residual departure	0.057	3
CRF ~ GMC_z	0.369	0.349	1.35	0.014	Mild residual departure	0.067	9
Flexibility ~ GMC_z	0.119	0.092	1.35	0.015	No important departure	0.306	4
Speed/Agility ~ GMC_z	0.327	0.306	1.35	0.090	Mild residual departure	0.269	10
GPF ~ PS_z	0.464	0.448	1.26	0.357	No important departure	0.118	5
LLMS ~ PS_z	0.397	0.378	1.26	0.235	Mild residual departure	0.057	3
CRF ~ PS_z	0.353	0.332	1.26	0.005	Mild residual departure	0.047	7
Flexibility ~ PS_z	0.114	0.086	1.26	0.017	No important departure	0.298	4
Speed/Agility ~ PS_z	0.296	0.274	1.26	0.249	Notable residual departure	0.222	7
GMC_z ~ DQI	0.241	0.217	1.12	0.171	Notable residual departure	0.097	9
GPF ~ DQI	0.342	0.321	1.12	0.744	No important departure	0.165	4
GPF ~ DQI + GMC_z	0.500	0.480	1.36	0.254	No important departure	0.106	6

GPF, Global Physical Fitness; LLMS, Lower-Limb Muscular Strength; CRF, Cardiorespiratory Fitness; GMC_z, product-oriented motor competence z-score; PS_z, process-oriented motor competence z-score; DQI, Diet Quality Index; VIF, Variance Inflation Factor. Residual assessment was based on residual-versus-fitted plots, Q–Q plots, Shapiro–Wilk tests, and Jarque–Bera tests. Values in bold indicate statistically significant Breusch–Pagan tests ($p < 0.05$). Sensitivity analyses using HC3 robust standard errors and exclusion of potentially influential observations are presented in Supplementary Table S5.

Table S6. Sensitivity analyses of adjusted multiple linear regression models using HC3 robust standard errors and influential-observation diagnostics.

Model	HC3 sensitivity	Influence analysis	Conclusion
CRF ~ GMC_z	No change	No material influence	Robust
Flexibility ~ GMC_z	No change	No material influence	Robust
CRF ~ PS_z	No change	No material influence	Robust
Flexibility ~ PS_z	No change	No material influence	Robust
Speed/Agility ~ PS_z	No change	Association lost significance after excluding influential observations	Interpret cautiously (influence-sensitive)

CRF, Cardiorespiratory Fitness; GMC_z, product-oriented motor competence z-score; PS_z, process-oriented motor competence z-score; HC3, heteroscedasticity-consistent (HC3) robust standard errors. Influence analyses were performed after excluding observations with Cook's distance > 4/n. No change indicates that the magnitude, direction, and statistical significance of the association remained materially unchanged after sensitivity analyses. Robust indicates that the primary conclusions were unchanged following both HC3 adjustment and influential-observation analyses. Interpret cautiously indicates that the association became non-significant after exclusion of potentially influential observations despite remaining significant when HC3 robust standard errors were applied.

STROBE Statement—Checklist of items that should be included in reports of ***cross-sectional studies***

	Item No	Recommendation	Page N°
**Title and abstract**	1	(*a*) Indicate the study's design with a commonly used term in the title or the abstract	1
		(*b*) Provide in the abstract an informative and balanced summary of what was done and what was found	2
**Introduction**			
Background/rationale	2	Explain the scientific background and rationale for the investigation being reported	2
Objectives	3	State specific objectives, including any prespecified hypotheses	3
**Methods**			
Study design	4	Present key elements of study design early in the paper	3
Setting	5	Describe the setting, locations, and relevant dates, including periods of recruitment, exposure, follow-up, and data collection	3
Participants	6	(*a*) Give the eligibility criteria, and the sources and methods of selection of participants	4
Variables	7	Clearly define all outcomes, exposures, predictors, potential confounders, and effect modifiers. Give diagnostic criteria, if applicable	5
Data sources/ measurement	8*	For each variable of interest, give sources of data and details of methods of assessment (measurement). Describe comparability of assessment methods if there is more than one group	*6*
Bias	9	Describe any efforts to address potential sources of bias	5
Study size	10	Explain how the study size was arrived at	4
Quantitative variables	11	Explain how quantitative variables were handled in the analyses. If applicable, describe which groupings were chosen and why	6
Statistical methods	12	(*a*) Describe all statistical methods, including those used to control for confounding	7
		(*b*) Describe any methods used to examine subgroups and interactions	7
		(*c*) Explain how missing data were addressed	7
		(*d*) If applicable, describe analytical methods taking account of sampling strategy	7
		(*e*) Describe any sensitivity analyses	7
**Results**			
Participants	13*	(a) Report numbers of individuals at each stage of study—eg numbers potentially eligible, examined for eligibility, confirmed eligible, included in the study, completing follow-up, and analysed	8
		(b) Give reasons for non-participation at each stage	8
		(c) Consider use of a flow diagram	8
Descriptive data	14*	(a) Give characteristics of study participants (eg demographic, clinical, social) and information on exposures and potential confounders	8
		(b) Indicate number of participants with missing data for each variable of interest	8
Outcome data	15*	Report numbers of outcome events or summary measures	9

Main results	16	(*a*) Give unadjusted estimates and, if applicable, confounder-adjusted estimates and their precision (eg, 95% confidence interval). Make clear which confounders were adjusted for and why they were included	10
		(*b*) Report category boundaries when continuous variables were categorized	11
		(*c*) If relevant, consider translating estimates of relative risk into absolute risk for a meaningful time period	11
Other analyses	17	Report other analyses done—eg analyses of subgroups and interactions, and sensitivity analyses	11
**Discussion**			
Key results	18	Summarise key results with reference to study objectives	11
Limitations	19	Discuss limitations of the study, taking into account sources of potential bias or imprecision. Discuss both direction and magnitude of any potential bias	13
Interpretation	20	Give a cautious overall interpretation of results considering objectives, limitations, multiplicity of analyses, results from similar studies, and other relevant evidence	12
Generalisability	21	Discuss the generalisability (external validity) of the study results	12
**Other information**			
Funding	22	Give the source of funding and the role of the funders for the present study and, if applicable, for the original study on which the present article is based	14

*Give information separately for exposed and unexposed groups.

Note: An Explanation and Elaboration article discusses each checklist item and gives methodological background and published examples of transparent reporting. The STROBE checklist is best used in conjunction with this article (freely available on the Web sites of PLoS Medicine at http://www.plosmedicine.org/, Annals of Internal Medicine at http://www.annals.org/, and Epidemiology at http://www.epidem.com/). Information on the STROBE Initiative is available at www.strobe-statement.org.
